# Video Segmentation of Wire + Arc Additive Manufacturing (WAAM) Using Visual Large Model

**DOI:** 10.3390/s25144346

**Published:** 2025-07-11

**Authors:** Shuo Feng, James Wainwright, Chong Wang, Jun Wang, Goncalo Rodrigues Pardal, Jian Qin, Yi Yin, Shakirudeen Lasisi, Jialuo Ding, Stewart Williams

**Affiliations:** 1 Welding and Additive Manufacturing Centre, School of Aerospace, Transport and Manufacturing, Cranfield University, Bedford MK40 3AA, UK; shuo.feng@cranfield.ac.uk (S.F.); j.c.wainwright@cranfield.ac.uk (J.W.); chong.wang1@cranfield.ac.uk (C.W.); jun.wang.123@cranfield.ac.uk (J.W.); g.pardal@cranfield.ac.uk (G.R.P.); s.williams@cranfield.ac.uk (S.W.); 2 WAAM3D Limited, Thornton Chase, Linford Wood, Milton Keynes MK14 6FD, UK; y.yin@waam3d.com (Y.Y.); s.lasisi@waam3d.com (S.L.); jialuo@waam3d.com (J.D.)

**Keywords:** wire + arc additive manufacturing (WAAM), video segmentation, deep learning, droplet transfer behaviour

## Abstract

Process control and quality assurance of wire + arc additive manufacturing (WAAM) and automated welding rely heavily on in-process monitoring videos to quantify variables such as melt pool geometry, location and size of droplet transfer, arc characteristics, etc. To enable feedback control based upon this information, an automatic and robust segmentation method for monitoring of videos and images is required. However, video segmentation in WAAM and welding is challenging due to constantly fluctuating arc brightness, which varies with deposition and welding configurations. Additionally, conventional computer vision algorithms based on greyscale value and gradient lack flexibility and robustness in this scenario. Deep learning offers a promising approach to WAAM video segmentation; however, the prohibitive time and cost associated with creating a well-labelled, suitably sized dataset have hindered its widespread adoption. The emergence of large computer vision models, however, has provided new solutions. In this study a semi-automatic annotation tool for WAAM videos was developed based upon the computer vision foundation model SAM and the video object tracking model XMem. The tool can enable annotation of the video frames hundreds of times faster than traditional manual annotation methods, thus making it possible to achieve rapid quantitative analysis of WAAM and welding videos with minimal user intervention. To demonstrate the effectiveness of the tool, three cases are demonstrated: online wire position closed-loop control, droplet transfer behaviour analysis, and assembling a dataset for dedicated deep learning segmentation models. This work provides a broader perspective on how to exploit large models in WAAM and weld deposits.

## 1. Introduction

### 1.1. The Demand for Automated Segmentation Method in WAAM and Welding

Wire + arc additive manufacturing (WAAM), also known as wire–arc directed energy deposition (arc-DED), is one of the most efficient metal additive manufacturing processes [1,2,3]. WAAM has distinct advantages in building large metal components with varying geometries. In the past decade, both academia and industry have actively pursued the commercialisation of WAAM techniques. However, the commercial adoption of WAAM technology is still in its infancy. Compared with proven methods such as forging and machining, there are still many process and quality control challenges in WAAM due to the complexity of WAAM techniques and the demanding part qualification standards in industries such as aerospace [4,5,6].

WAAM systems may use a gantry system or robotic arm fitted with different power sources, such as tungsten inert gas (TIG), plasma-transferred arc (PTA), and metal inert gas welding (MIG). Various cameras and sensors such as optical cameras, thermal cameras, laser scanners, and pyrometry, and gas, voltage, and current sensors, etc., are installed in the systems to monitor the deposition process [4,7,8,9].

Vision monitoring plays a pivotal role in ensuring the quality and efficiency of WAAM processes [10,11,12]. By providing real-time visual data, it enables operators to monitor melt pool geometry, arc stability, and wire feeding stability. Real-time visual data can also be used for defect detection, e.g., porosity and cracks, geometric deviations and layer quality problems. Vision systems can provide real-time feedback to the control system, allowing for immediate adjustments to process parameters as necessary [13,14]. This helps maintain a stable and consistent deposition process.

The role of the WAAM operator is highly demanding, as it requires continuous monitoring of live video feeds for several hours and rapid identification and response to anomalies within seconds. The optimal way to implement process control in WAAM is to use machine vision algorithms to segment and quantify videos and apply the results in closed-loop control.

### 1.2. Challenges of Segmenting Welding Images and Videos

Segmenting WAAM images and videos, i.e., the process of identifying specific regions or objects within the visual data, presents several unique challenges due to the nature of WAAM processes and the characteristics of the resulting imagery.

WAAM often involves moving parts, arcs, fumes, spatters, and metal transfer creating rapidly changing visual patterns. Melt pools, base materials, end effector, and embedded sensors can occlude one another, making it difficult to identify their complete boundaries. The uneven distribution of bright arc can create shadows, making it challenging to accurately identify object boundaries. The WAAM equipment, equipment safety enclosure, and the built part can reflect light, causing glare and obscuring details. The surface condition of the built part and baseplate, such as slag or oxidation scale, can affect their visual appearance and segmentation accuracy.

Conventional segmentation algorithms, such as region-based methods, edge detection, thresholding, and clustering, are often limited to specific ideal conditions. Their performance can degrade significantly when confronted with varying light levels, material changes, or other parameter fluctuations. Moreover, these traditional methods frequently necessitate manual specification of the region of interest (ROI) and parameter tuning, which can be both time-consuming and prone to errors [15].

Deep learning models, such as U-Net [16,17,18,19,20], Mask R-CNN [21], and DeepLab [22], present a promising approach to WAAM video segmentation. These models, capable of learning complex patterns directly from data, offer the potential to overcome the limitations of traditional segmentation methods. However, a significant barrier to their widespread adoption is the substantial time and resource investment required to construct large, high-quality datasets with accurate annotations, tailored specifically to the unique characteristics of WAAM processes [15].

The Segment Anything Model (SAM) [23,24,25] has recently gained substantial recognition as a foundational model in computer vision. Trained on a natural image dataset of over 1 billion masks, the SAM demonstrates exceptional proficiency in generating precise object masks from various prompts, including bounding boxes, points, and text, or even autonomously. However, the SAM’s applicability to specialised domains like medical imaging [24,26,27], welding, or WAAM is often limited by the significant differences between these image types [28]. While natural images typically feature objects with clear boundaries, target objects in WAAM images often have less distinct edges. To address this, researchers often need to fine-tune the SAM using their specific datasets [26,27,29].

Semantic segmentation involves assigning a semantic label to each pixel in an image. When applied to videos, this involves labelling each pixel in every frame with a corresponding object category. Video semantic segmentation can be achieved by applying segmentation models to each frame individually or exploiting semi-supervised fine-grained object tracking model initialised with specific object mask ground truth or using interactive video object segmentation tool.

The Track Anything Model (TAM) [30] and Segment and Track Anything Model (SAM-Track) [31] use the Gradio package [32] to create an interactive user interface (UI). They leverage user-provided prompts and the SAM to perform semantic segmentation on the first frame of a video. Subsequently, object tracking models, e.g., XMem [33], Cutie [34], and DeAOT [35], are utilised to segment the remaining frames. In cases where the object tracking model yields unsatisfactory results, the SAM can be used for refinement, or the user can terminate the process. However, when applying those models directly to WAAM and welding videos, the results are not satisfactory. This is because the SAM struggles to accurately segment objects with blurred boundaries, such as the melt pool edge and molten droplets, etc.

In this study, a semi-automatic segmentation tool for WAAM videos was developed based on the computer vision foundation model SAM and the video object tracking model XMem. To facilitate segmentation of WAAM videos, the tools are extended with functionalities including video cropping and editing, manual annotation, and bidirectional object tracking. This work provides a rapid and reliable video segmentation tool specifically for WAAM and welding; it also introduces a fast method for assembling video datasets. This work broadens the perspective of utilising AI large models to address challenges in WAAM and welding.

## 2. Methodology

### 2.1. Pipeline of WAAM Videos Segmentation

**Figure 1:**
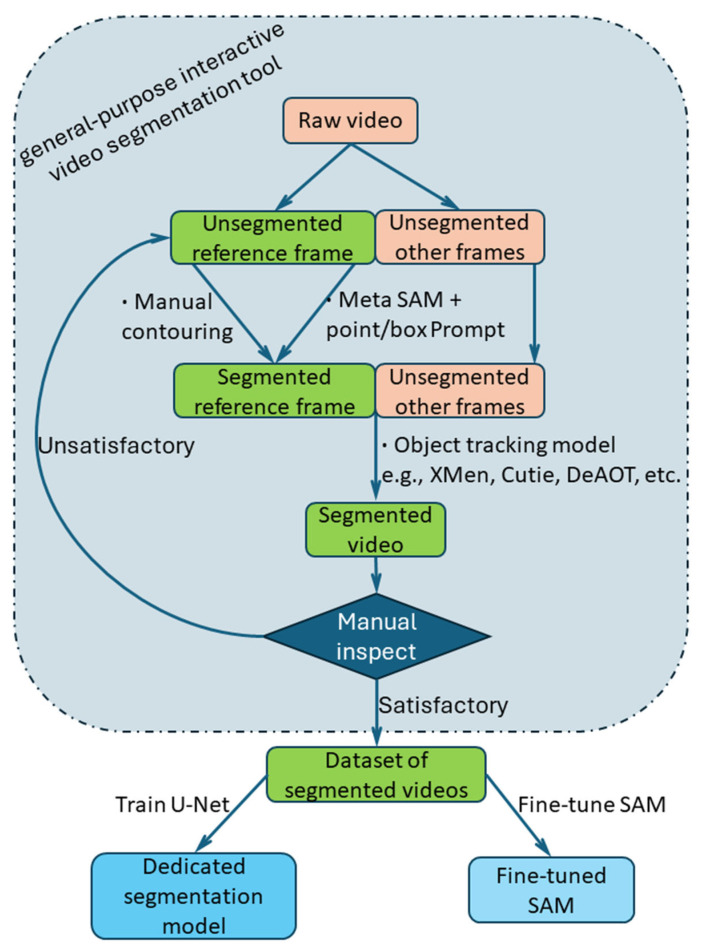
The pipeline of our work on WAAM video segmentation.

Figure 1 illustrates the process flow for this investigation of WAAM video segmentation. The following sections introduce the toolkit, which is based on the SAM (ViT-H) foundation model and the XMem object tracking model. Three cases are given to demonstrate the effectiveness of the tool.

The toolkit can be used for in-process wire tip position feedback control. Based on the results of offline WAAM video segmentation, the trend of droplet cross-sectional area versus time was plotted, and the number and frequency of droplets were measured. When higher segmentation speed is required, the toolkit can be used for assembling large datasets for dedicated deep learning segmentation models. The assembled WAAM dataset can then also be used to fine-tune the SAM.

### 2.2. The Toolkit for WAAM Video Segmentation

**Figure 2:**
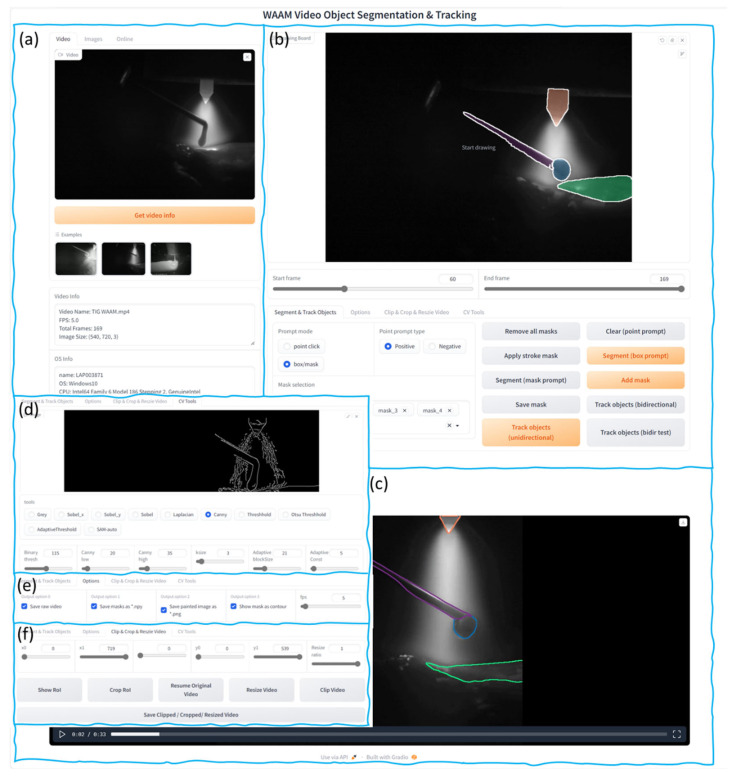
UI of the general interactive tool for WAAM video analysis. (**a**) Video import section, (**b**) reference frame segmentation tools and tracking model options, (**c**) video segmentation results, (**d**) computer vision tools area, (**e**) segmentation result saving options, (**f**) video clipping and editing tools.

Gradio is a Python v.3.11 library designed to create interactive user interfaces (UI) for machine learning (ML) models. It enables the rapid development of user-friendly web interfaces for models. Gradio supports a wide range of input and output types, e.g., number, text, images, videos, and audio. Once a Gradio application is created and launched, it starts a local web server and generates a uniform resource locator (URL). This URL can then be opened in a web browser to interact with the model. The WAAM video segmentation toolkit UI is constructed using the Gradio library. Figure 2 demonstrates the UI created for this study. The toolkit is mainly used for offline WAAM video segmentation. Functionally, the interface is divided into several regions. Figure 2a demonstrates the video import section, where videos can be imported either by drag-and-drop or by selecting the video’s file path. After video import, video name, the total number of frames, frame rate, and resolution of the video, as well as the computer’s OS information, CPU, GPU, and memory usage are displayed in this area. Figure 2b is the keyframe (or reference frame) segmentation and video object tracking options area. The keyframe segmentation options include SAM’s point prompt mode, box/mask prompt mode, and manual contouring of target objects.

Given the subpar performance of SAM’s text prompt mode, this option was excluded from this tool. The video object tracking options include unidirectional tracking and bidirectional tracking, Figure 2c is the result display area after video segmentation. Figure 2d is the computer vision tools area, where various operations can be performed on a selected frame, such as Sobel operator, Canny operator, Otsu thresholding, adaptive thresholding, and SAM automatic segmentation, etc. The results are displayed directly in this area. Figure 2e is the video segmentation result saving options area, where users can choose whether to save the mask results of each frame and modify the frame rate of the segmented video. Figure 2f is the video clipping and editing tools area, where users can zoom in, zoom out, crop, and clip the original video.

### 2.3. Comparison of Segmentation Methods

**Figure 3:**
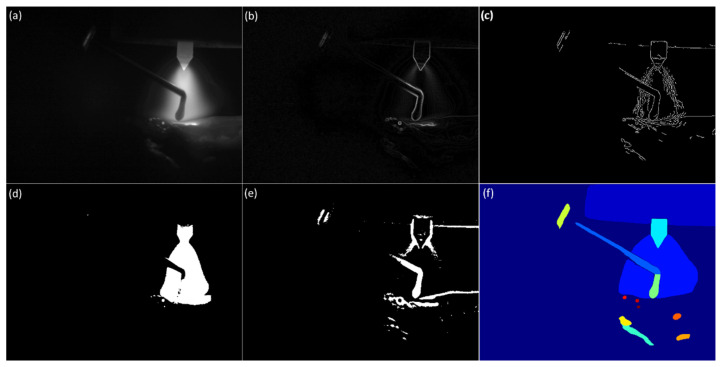
(**a**) Clear frame from a WAAM monitoring video, applying the (**b**) Sobel algorithm, (**c**) Canny algorithm, (**d**) Otsu thresh algorithm, (**e**) Adaptive thresh algorithm, and (**f**) Meta SAM to the frame.

**Figure 4:**
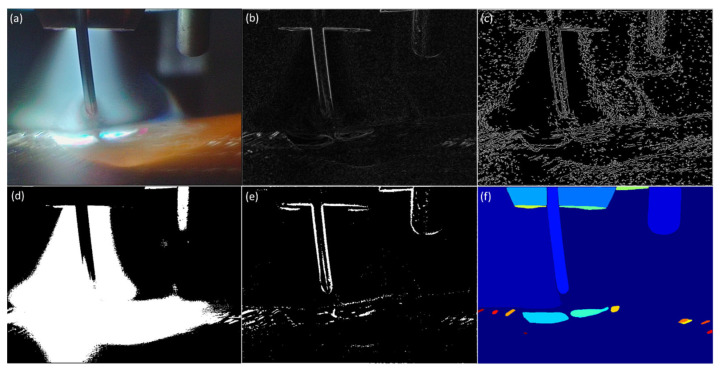
(**a**) Blur frame from a WAAM monitoring video, applying the (**b**) Sobel algorithm, (**c**) Canny algorithm, (**d**) Otsu thresh algorithm, (**e**) Adaptive thresh algorithm, and (**f**) Meta SAM to the frame.

The developed interactive video segmentation toolkit allows users to preview the segmentation results of various computer vision algorithms before performing segmentation. Figure 3 and Figure 4 present the results of applying the most common algorithms to a frame from a clear video and a frame from a blurry video, respectively. It is worth noting that both clear and blurry frames typically coexist within a single video due to the presence of multiple moving objects during the WAAM and welding processes.

This is a significant reason why automatic segmentation of WAAM videos is challenging. Figure 3b and Figure 4b demonstrate the results of the Sobel gradient operator. Figure 3c and Figure 4c display the results of the Canny edge detector. Figure 3c displays the characteristics of unclear and discontinuous boundaries in WAAM videos. Figure 4c demonstrates the characteristics of strong noise captured in WAAM videos. Figure 3d and Figure 4d show the results of Otsu’s thresholding. Figure 3e and Figure 4e show the results of the adaptive thresholding algorithm. The automatic segmentation results produced by the foundational machine vision model SAM, as illustrated in Figure 3f and Figure 4f, reveal its inability to effectively segment blurry objects like melt pool boundaries and droplets in WAAM videos.

### 2.4. Annotate One Key Frame

This section demonstrates the use of an interactive segmentation tool to annotate a keyframe or reference frame and then utilise a video object tracking model to segment the remaining frames. The first step of segmentation is to select a specific frame as the keyframe or reference frame. The keyframe is required to include all objects desired to segment, such as electrode, arc, feeding stock (wires), droplets, and melt pools. Additionally, the keyframe should be one of the clearest frames among all frames, which is beneficial for segmentation.

**Figure 5:**
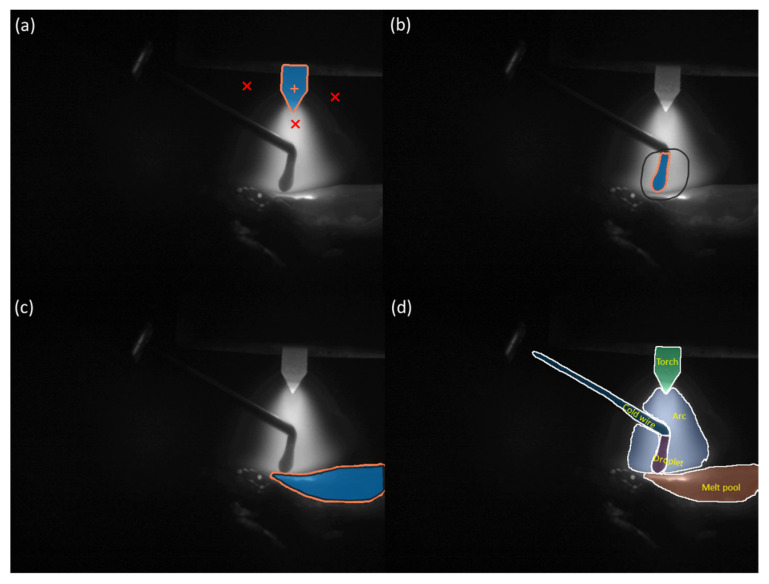
(**a**) Annotates a frame with point prompt mode: ‘+’ indicates a positive prompt; ‘×’ indicates a negative prompt. (**b**) Annotates a frame with box prompt mode. (**c**) Annotates a frame with manual contouring target object through mouse dragging or drawing tablets input. (**d**) An annotated reference frame, the starting point for segmentation of the remaining frames.

Figure 5a illustrates the process of segmenting target objects using the point prompt mode of the SAM. In this mode, segmentation is achieved by clicking on the target object with a mouse. Each mouse click updates the segmentation result. The pixels where the mouse is clicked are displayed with either a ‘+’ or ‘×’ symbol. A ‘+’ indicates a positive prompt, meaning that the point belongs to the target object. An ‘×’ indicates a negative prompt, meaning that the point does not belong to the object to be segmented. While objects with distinct boundaries, like tungsten electrodes, can be accurately segmented with one or a few clicks, objects with blurred boundaries, such as droplets and wires, may require multiple clicks or even fail to segment.

When the point prompt mode fails to yield a satisfactory segmentation result after multiple attempts, the box/mask prompt mode can be utilised. In this mode, a polygonal region of interest can be defined by dragging the mouse cursor. The SAM then performs segmentation based on this region of interest. Figure 5b,c present an example of segmenting a droplet using the box selection mode. Experience suggests that tasks achievable through point prompt mode can also be accomplished using box selection mode. If a segmentation task cannot be completed even with box selection or mask refinement, it indicates that the task exceeds the capabilities of the SAM, necessitating manual contouring of the target object, shown in Figure 5d.

When manually outlining the target, a high-precision input tool such as a drawing tablet can be used to delineate the object’s contour. The manually outlined contour will be directly used as the target mask without being provided to the SAM for mask prediction.

After segmenting a target object, clicking the “Add mask” button in the UI saves the segmentation result and allows selection of the next object segmentation. Once all objects have been segmented, a multi-object tracking model can be employed to segment the entire video. Following video segmentation, operators can review frame-by-frame results in the user interface or in the default saved results folder. If the results are satisfactory, the video segmentation process can be repeated for the next video.

When the results are completely unacceptable (which is not common, such as when errors occur within one or a few frames of the keyframe), the keyframe can also be reselected and the video can be re-segmented. A common problem encountered is that as the number of tracked frames increases (e.g., after several hundred frames from the keyframe), the segmentation results for one or more objects may become unacceptable. This is because the object tracking models, which simulate the brain’s memory mechanism, need to frequently update the feature information or a template of the tracked target. Once an error occurs (which is common in videos with poor quality or blurry objects), the tracking model’s prediction errors may gradually accumulate. In such cases, the correctly segmented portion of the video can be clipped, and the remaining portion can be re-segmented. This allows for segmenting a long video of several thousand or tens of thousands of frames by dividing it into multiple shorter segments. Alternatively, the entire video can be re-segmented without updating the template. However, if the video quality is good and the target objects are clear, a single keyframe can be used to segment the entire video.

Video segmentation is essential for quantitative video analysis and vision-based closed-loop control. Once video segmentation is complete, traditional machine vision algorithms can be readily employed to quantitatively analyse the segmented object’s attributes (e.g., size, shape, or position). By segmenting video streams in real-time and calculating the attributes and adjustment values of the regulated object, these values can be fed into the actuator, enabling vision-based closed-loop control.

## 3. Case Studies

### 3.1. Wire Position Closed-Loop Control

Real-time monitoring and closed-loop control of the droplet transfer mode are essential for ensuring the stability of the WAAM process. Rios et al. [10] identified three transfer modes (TMs): TM1, surface tension transfer, where the wire is in constant contact with the melt pool; TM2, the preferred method of stable droplet transfer, where small consistent droplets are generated; and TM3, a form of globular transfer which is unstable and reliant on the formation of large free-falling droplets. The wire feeding position, as identified by Wang et al. [36], is a crucial parameter influencing both the transfer mode and overall process stability. Operators typically adjust the wire feed position manually based on real-time process camera images to maintain a stable TM2 transfer mode. However, this manual intervention can be labour-intensive and unrealistic, considering the potentially long (hours) duration of the WAAM process. To address these challenges, online monitoring and closed-loop control systems offer a more effective and convenient solution. While arc voltage monitoring can be used for closed-loop control with certain power sources, real-time video monitoring from a process camera provides a more intuitive and direct approach across all.

**Figure 6:**
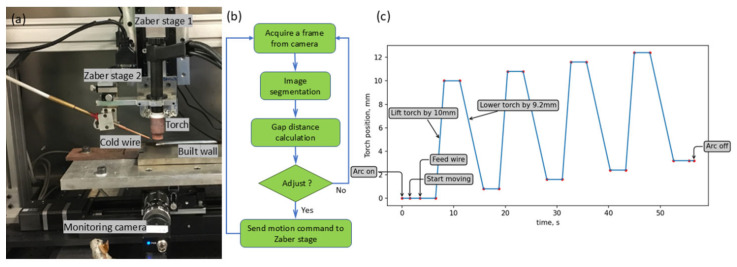
(**a**) Experiment configuration. (**b**) The workflow of wire position close-loop control. (**c**) Predefined vertical motion profiles of torch and wire.

Figure 6a shows the WAAM system used for the wire position closed-loop control testing. The computer numerical control (CNC) WAAM system consists of four Zaber stages, a TIG power source (EWM Tetrix 230 plus), TIG torch, wire feeder, and an industrial (Hikrobotics MV-CA016-10UM) camera. The monochrome industrial camera employed for our WAAM deposition monitoring has a resolution of 720 × 540 pixels, a Ricoh lens of 25 mm focal length, and is positioned ~150 mm from the observation target. For the system used, Zaber stage 1 is responsible for the vertical (*Z*-axis) movement of the TIG torch and cold wire. Zaber stage 2 is an additional small motorised linear stage (maximum stroke of 10 mm) and is responsible for adjusting the height of the wire in the Z direction with respect to the torch.

The other Zaber stages are responsible for the horizontal movement (X and Y axes) of the deposited part. Zaber stage motion was controlled by a computer using a Python script. The filler wire used was low-carbon steel of diameter 1.2 mm. The experiment was conducted with a wire feed speed of 2.2 m/min and a deposition travel speed of 4 mm/s; a welding current of 190A was used to deposit a wall of 200 mm length.

Figure 6b shows the flowchart for the closed-loop control of the cold wire position. The process begins by capturing a real-time image from the process camera, which is then subjected to video segmentation. The segmented image is analysed to determine the distance between the cold wire tip and the melt pool. This measured distance is compared to the desired distance, and the discrepancy is used to calculate the necessary adjustment. The calculated adjustment value is then transmitted to Zaber stage 2, which moves vertically to maintain the desired distance between the cold wire tip and the melt pool.

To assess the performance of our proposed closed-loop system, the CNC WAAM system was subjected to a controlled experiment. Zaber stage 1 was programmed to execute a predefined vertical motion profile, as illustrated in Figure 6c, which resulted in changes to the positions of both the TIG torch and the cold wire. Simultaneously, the closed-loop control programme adjusted Zaber stage 2 in response to real-time image feedback, thereby maintaining a constant distance between the cold wire tip and the melt pool. After the deposition starts, Zaber stage 1 will lift the torch and the cold wire first, remain stationary for 4 s, then proceed downward and remain stationary for a further 4 s. This intentional up-and-down process was repeated four times.

**Figure 7:**
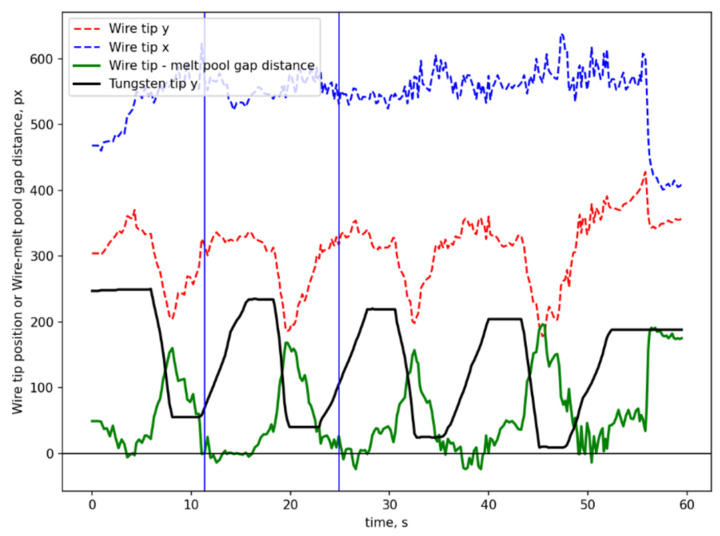
Cold wire position, tungsten electrode tip position, and the distance between the cold wire and the melt pool in wire position closed-loop control.

**Figure 8:**
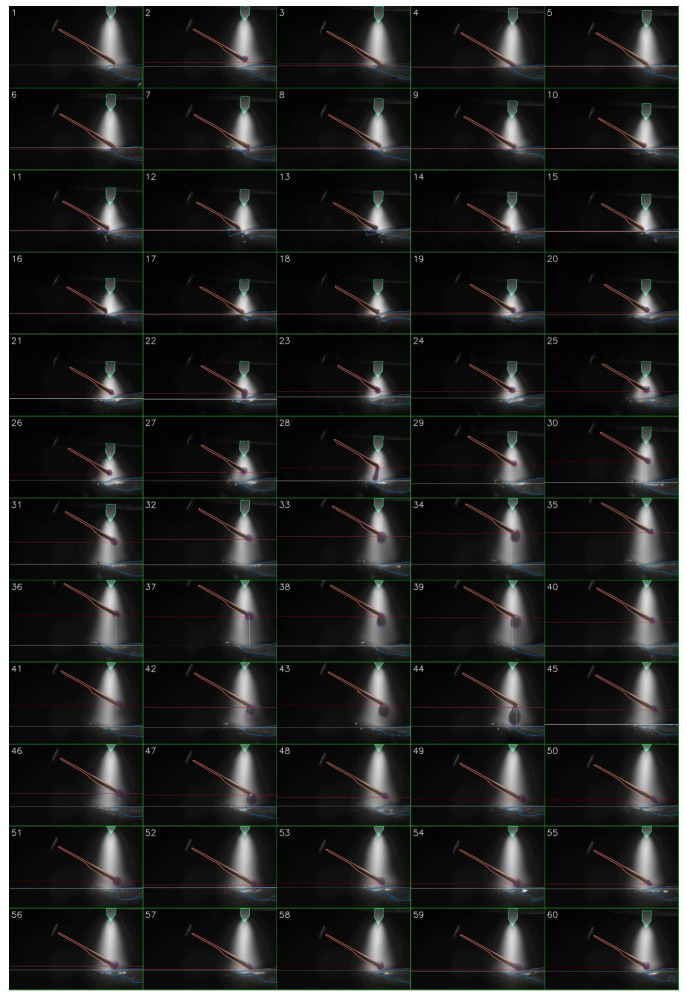
A sequence of 60 frames from the closed-loop control video. The horizontal red line represents the cold wire height position, and the horizontal white line represents the melt pool position.

Figure 7 presents the curves representing the cold wire position, tungsten electrode tip position, and the distance between the cold wire and the melt pool during the vision-based closed-loop control of the cold wire position. Figure 8 demonstrates a part of the closed-loop control video, which consists of 60 consecutive frames. The time interval corresponding to Figure 8 is between the two blue vertical lines in Figure 7. These results demonstrate the effectiveness of the closed-loop control system for regulating the cold wire position. Figure 7 shows that there is a certain degree of hysteresis in the adjustment of the distance between the cold wire and the melt pool. This is because the cold wire undergoes a large position change in a very short time (e.g., rising or falling 10 mm in about 1 s). Zaber stage 1 can rise or fall at a speed of 9 mm/s, while the maximum speed of Zaber stage 2 can only reach 2 mm/s. Since such extreme conditions are not representative of typical WAAM operating conditions and Zaber stage 2 can be replaced with a faster stage, this hysteresis will not cause a problem. To be highlighted, the wire position control function in this paper is only validated by a fixed set of process parameters. More comprehensive wire position control study should be performed with the support of the proposed segmentation. The full video is available in Supplementary Materials.

### 3.2. Droplet Transfer Behaviour Analysis

Droplet transfer, the process by which molten metal is transferred from the electrode or filler wire to the workpiece in welding and WAAM, is a critical factor influencing weld and build quality, efficiency, and defect generation [24]. Understanding droplet transfer is essential for optimising the process and achieving desired results. High-speed cameras and other imaging techniques have been used to study droplet transfer. These visualisations provide valuable insights into the dynamics of the process and help researchers develop models and improve welding practices. Droplet transfer dynamics involve droplet formation, droplet detachment, droplet flying trajectory and droplet impact. To characterise the process of material droplet feeding into the melt pool, four droplet transfer modes were identified for plasma and TIG processes: permanent contact mode, intermittent contact mode, fleeting contact mode, and non-contact mode [24]. These modes are categorised based on the duration of droplet formation and the duration of contact between the droplet and the melt pool.

The equipment used for this research is similar to that in wire position closed-loop control case: the CNC WAAM system consists of three Zaber stages, a plasma power source, a plasma torch, a wire feeder, and an industrial camera (Hikrobotics MV-CA016-10UM, Hangzhou, China). Three Zaber motorised linear stages align along the X, Y, and Z axes. Their motion was controlled by computer using a Python script. The plasma power source consists of a Pettenbach, Austria DC power source and FRONIUS Plasma Module 10. The filler wire was low-carbon steel with a diameter of 1.2 mm. The monochrome industrial camera employed for our WAAM deposition monitoring has a resolution of 720 × 540 pixels, a Ricoh lens of 25 mm focal length, and is positioned approximately 250 mm from the observation target. The camera operates at a frame rate of 318 frames per second, which is sufficient to fully capture the plasma WAAM droplet transfer process. The experiment was conducted with a wire feed speed of 2.2 m/min, a plasma torch speed of 4 mm/s, and a welding current of 180 A.

**Figure 9:**
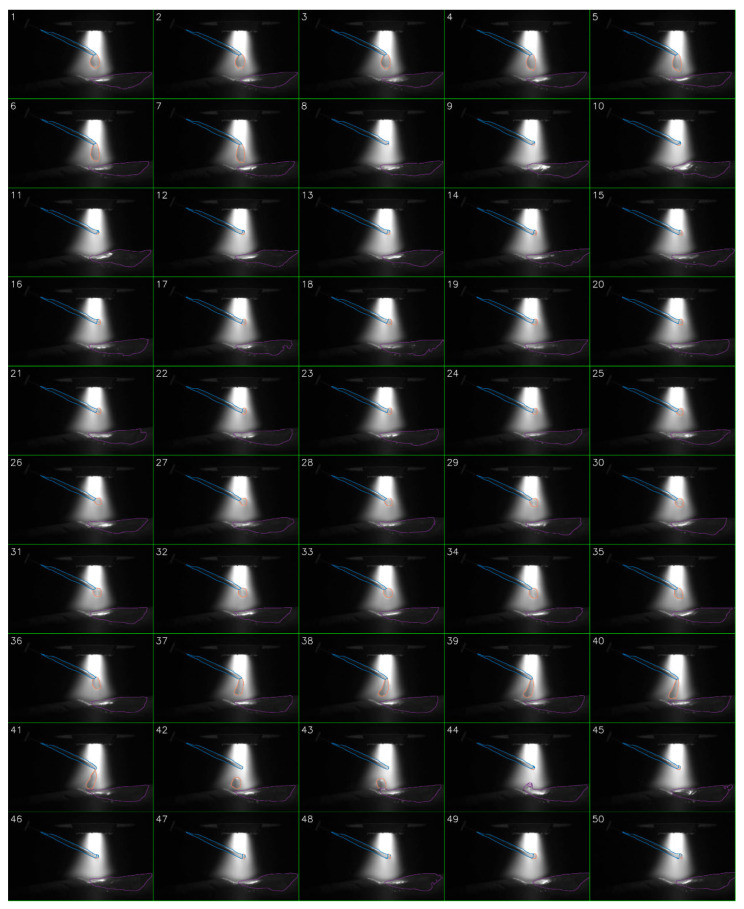
A 50-frame sequence extracted from a segmented plasma WAAM video, showcasing a complete droplet transfer process.

**Figure 10:**
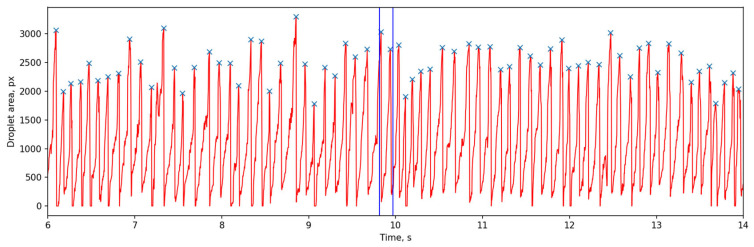
The droplet cross-sectional area (in pixels) over time.

Figure 9 presents a 50-frame sequence extracted from a segmented monitoring video using our toolkit. The entire video, spanning ~17.8 s and 5660 frames, captures a single pass of the plasma deposition process. Subfigures 8 to 44 in Figure 9 illustrate the complete process of a droplet from its formation, growth, detachment, descent, and entry into the melt pool. The full video is available in Supplementary Materials. Figure 10 plots the droplet cross-sectional area (in pixels) over time. The maximum droplet cross-sectional area prior to melt pool entry is marked with an ‘×’ on the curve. Statistical analysis reveals 155 droplets in total during this process, with an average droplet falling frequency of 8.7 Hz. The time interval corresponding to Figure 9 is indicated between the two blue vertical lines in Figure 10.

### 3.3. Assemble a Dataset for Dedicated WAAM Segmentation Model

In the previous two cases, the segmentation model developed was run on a laptop equipped with an NVIDIA GeForce RTX 4090 mobile GPU with 16 GB (Santa Clara, CA, USA) of dedicated VRAM. When segmenting videos of 720 × 540 pixels, it can achieve a speed of 12–15 frames per second, and when segmenting videos of 1440 × 1080 pixels, it can only achieve a speed of 0.5–2 frames per second. Segmentation speed is less critical for offline applications. However, it is important in many cases when using the model in situ. A frame rate of 12–15 fps is adequate for closed-loop control of the cold wire position, but higher frame rates are required for droplet behaviour studies.

To accelerate the model, alternatives can be explored to the general object tracking model by adopting a faster dedicated segmentation model, along with leveraging a more powerful GPU. Nevertheless, training such a model necessitates a substantial dataset. Fortunately, the proposed tool is well-suited for generating the required dataset for a customised model.

A dataset of 30k images was curated from a collection of WAAM monitoring videos captured by XIRIS welding cameras (Xiris Automation Inc., Burlington, ON, Canada). The filler wire used was Titanium 64, with a diameter of 1.2 mm. The experiment was conducted with a wire feed speed of 1.6 m/min, a deposition travel speed of 3 mm/s, and a welding current of 160 A to deposit a wall 200 mm in length. These videos, ranging from hundreds to thousands of frames, were segmented using the customised toolkit. Each frame was manually inspected to identify and correct segmentation errors, resulting in a high-quality dataset. Due to the high efficiency of the tool developed (as demonstrated in the previous two case studies), a large proportion of video segments can be correctly segmented without errors. The manual inspection of segmentation results is only a final confirmation. Therefore, assembling such a dataset takes approximately a single week for one person on a laptop with an NVIDIA GeForce RTX 4090 GPU.

The U-Net architecture was selected for a custom WAAM semantic segmentation model based on its proven effectiveness in image segmentation tasks and its computational efficiency [37]. The U-Net, known for its distinctive U-shaped topology, is a fully convolutional neural network composed of an encoding path and a decoding path. The encoding path, or contractive path, reduces the spatial dimensions and increases the number of feature channels through repeated convolutional layers, ReLU activations, and max pooling operations. The decoding path, or expansive path, up-samples the feature maps and combines them with corresponding features from the encoding path via skip connections to recover spatial information. The final output is a pixel-wise segmentation map.

To alleviate the problem of limited training data, transfer learning was adopted by utilising pre-trained models on large-scale datasets for other computer vision tasks [38]. Specifically, the encoder was replaced of our U-Net with a ResNet-50 backbone pre-trained on ImageNet. ResNet (a deep residual network) employs identity skip connections to mitigate the degradation problem that occurs when increasing the network depth. The encoder of the U-Net was frozen to preserve its learned features, while the decoder was fine-tuned to adapt to the new task.

**Figure 11:**
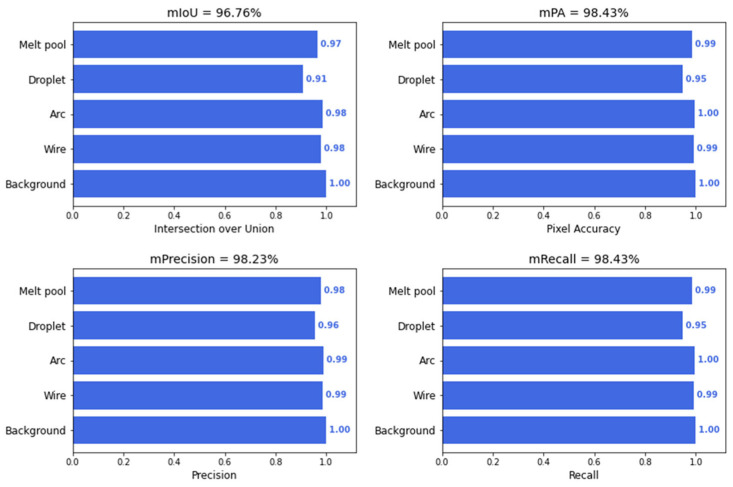
The segmentation metrics of trained U-net on the test dataset.

The model was developed using PyTorch v.2.7.0 and trained on a laptop with an Intel i9-13900H CPU and an NVIDIA GeForce RTX 4090 GPU. Training was performed using an input image size of 640 × 512 pixels, a batch size of 8, and a learning rate of 0.001. The model was trained for 120 epochs using the Adam optimizer and the Dice loss function. The training process took approximately 20 h. The trained model achieved a mean F1 score of over 0.95 and a mean Intersection over Union (mIoU) of over 0.96 on the test set (10% of the dataset). The metrics of trained U-net on the test dataset are shown in Figure 11.

**Figure 12:**
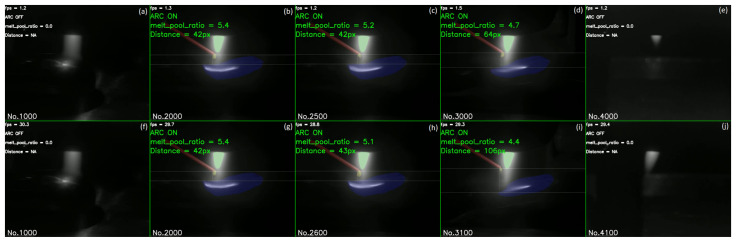
Dedicated model results on CPU (**a**–**e**) and GPU (**f**–**j**). The horizontal solid lines represent the cold wire height position and the melt pool position. The frame number of the current video frame is displayed in the bottom left corner. Video processing speed, arc status, the ratio of the molten pool cross-sectional area to the total image area and the distance between the cold wire and the molten pool are shown on top left.

Figure 12 demonstrates the generalisation ability of our trained model on unseen videos. When running on an i9-13900H CPU, the model achieved a processing speed of 1.2–1.5 frames per second, as shown in Figure 12a–e. In contrast, when running on a GPU, the processing speed was significantly improved to 28.8–30.3 frames per second on average, as depicted in Figure 12f–j. This represents a twentyfold improvement over previous generic segmentation model. The full video is available in Supplementary Materials.

## 4. Discussion

The rise in large models and foundation models in language and vision fields has been significant in recent years. These advancements have fuelled the development of numerous AI or ML applications. While AI is undeniably powerful, integrating it into various industrial processes remains challenging. Since existing AI vision large models are primarily trained on natural images and lack industrial data like WAAM and welding images, directly applying them to industrial scenarios will not yield optimal results. However, drawing inspiration from medical image research, with slight adjustments and additional steps such as data pre-processing and data post-processing, as well as model fine-tuning, these AI large models can become powerful tools for various industrial branches. The bottleneck lies not in AI’s capabilities but in its integration. This paper explores the application of large models and foundation models to WAAM and welding video analysis, demonstrating that the customised toolkit based on large models and foundation models outperforms traditional machine vision in terms of accuracy, flexibility, reliability, and efficiency. The vision large model SAM requires manual input of point or box prompts and can only be used to segment objects with well-defined boundaries. Visual tracking models necessitate a segmented reference frame as an initial value to function. However, for most individuals who are not image processing specialists, manually segmenting an image is a cumbersome or even impossible task. Therefore, a user-friendly interface is needed to integrate these powerful models and accomplish tasks that individual large models cannot handle alone. Within the same user interface, various models and tools, including contouring tools and traditional vision models, can complement each other, enabling the application of AI large models in industrial scenarios.

There is no publicly available image or video segmentation dataset specifically for the welding and WAAM fields. This makes it impossible to quantitatively measure model performance, unlike in other machine vision domains. The current approach involves saving the segmentation results for each frame, with a human then manually determining whether to accept or reject them. This method is both fast and effective for practical applications. With the aid of this toolkit, manually segmenting a reference frame typically takes about ten seconds to one minute. Using this reference frame, hundreds or thousands of acceptable segmentation results can usually be obtained in approximately ten minutes. Before the development of this toolkit, manual segmentation was attempted. For example, two individuals could only complete approximately 4000 frames in about three weeks. This toolkit has been widely utilised for WAAM wire tip position control and data analysis. Its application has encompassed materials such as steel, titanium alloys, and nickel-based alloys, spanning a wide range of process parameters. In the next phase of work, it is planned to categorise and save the confirmed acceptable segmentation results based on various equipment and processes, process parameters, materials, observation angles, illumination, and so forth. Once sufficient data has been accumulated, this dataset can then serve as a quantitative benchmark.

Within this paper, the comparison of segmentation speed between the CPU and GPU was confined to the same laptop. The dependence of this toolkit’s segmentation speed on hardware configurations, such as the CPU and GPU, will be analysed in the next phase of work.

Since the video analysis tool developed in this research is built upon a general-purpose large model, its applications extend beyond WAAM and welding video segmentation. In fact, it can be applied to any sequence of images of the same type. For instance, whilst not reported here, it has been applied to CT image segmentation, where the parts or holes are annotated in a single image and then have the model track these parts or holes in subsequent images. This toolkit has also been applied to the analysis of images captured by long-wave and short-wave infrared cameras. Infrared imagery used for process monitoring in welding, additive manufacturing, repair, and related applications often contains information from multiple targets. Segmentation facilitates the processing of temperature data by isolating the temperature field of individual objects. Additionally, the toolkit can be used to analyse material microstructure images; a single image or multiple images can be cropped and converted into an image sequence following pre-processing.

One particularly intriguing aspect of AI is its ability to evolve through iteration. Initially, a weak model may produce inaccurate predictions. By identifying these errors, manually correcting them, and incorporating the revised data into the training dataset, the model’s performance can be enhanced. As the dataset grows and iterations increase, the model becomes increasingly robust. In future development, it is planned to fine-tune the SAM using the segmented dataset to create the WAAM–SAM or Welding–SAM. This will reduce the need for manual delineation of target contours and further improve the level of automation.

The findings in this work also hold strong potential for advancing in situ alloying strategies in WAAM for novel materials exploration, such as multi-wire configurations involving feedstocks of different compositions. These approaches are especially valuable for manufacturing hard-to-process alloys, such as intermetallic compounds or high-entropy alloys, which cannot be directly wire-drawn. By enabling precise real-time monitoring of droplet behaviour, arc stability, and melt pool dynamics, the segmentation technique supports better control of compositional mixing and phase formation during deposition. This contributes to a deeper understanding of how process conditions influence alloy homogeneity, phase evolution, and defect mitigation in novel material systems, ultimately facilitating the design of new WAAM-compatible materials.

Quantifying the melt pool and droplets in monitoring videos allows for a deeper understanding of the underlying physical mechanisms. In future work, this data can be leveraged for various applications, including comparison with computational fluid dynamics (CFD) and finite-element models (FEM) as well as training data-driven models for process optimisation.

## 5. Conclusions

This paper introduces a toolset designed to harness large vision models for WAAM and welding video analysis. The key findings are summarised as follows:

1. Large AI models intended for general purposes cannot be directly applied to specific industrial domains due to either the absence of relevant domain-specific data or inherent data limitations.
2. A user-friendly interface integrating large models facilitates the practical application of complex segmentation workflows, as demonstrated through case studies; however, further evaluation is required to quantify the benefits of model integration.
3. For WAAM videos, employing a custom segmentation tool for a reference frame, followed by an object tracking model for subsequent frames, provides a robust method for rapid and reliable video segmentation. This approach remains effective across various camera types, viewing angles, and environmental backgrounds.
4. The proposed methodology has been successfully applied in WAAM digitalisation case studies, including feedstock position control, droplet transfer mode analysis, and WAAM-specific model development.

## Data Availability

The original contributions presented in this study are included in the article/Supplementary Materials. Further inquiries can be directed to the corresponding author.

## Supplementary Materials

The following supporting information can be downloaded at: https://www.mdpi.com/article/10.3390/s25144346/s1, Video S1: Wire tip position control—video; Video S2: Droplet—video; Video S3: u_net model results—video.
